# Deep Eutectic Solvent Ultrasonic-Assisted Extraction of Polysaccharides from Red Alga *Asparagopsis taxiformis*: Optimization, Characterization, Mechanism, and Immunological Activity in RAW264.7 Cells

**DOI:** 10.3390/foods15030438

**Published:** 2026-01-25

**Authors:** Kun Yang, Yuxin Wang, Wentao Zou, Qin Liu, Riming Huang, Qianwang Zheng, Saiyi Zhong

**Affiliations:** 1 Shenzhen Research Institute, Guangdong Ocean University, Shenzhen 518108, China; yk128611@163.com; 2 Guangdong Provincial Key Laboratory of Aquatic Product Processing and Safety, Guangdong Province Engineering Laboratory for Marine Biological Products, Guangdong Provincial Engineering Technology Research Center of Seafood, Key Laboratory of Advanced Processing of Aquatic Product of Guangdong Higher Education Institution, College of Food Science and Technology, Guangdong Ocean University, Zhanjiang 524088, China; 18091425115@163.com (Y.W.); zwt2025501@163.com (W.Z.); 3 Centre for Chinese Medicine Drug Development Limited, Hong Kong Baptist University, Hong Kong SAR, China; qinliu@hkbu.edu.hk; 4 College of Food Science, South China Agricultural University, Guangzhou 510642, China; huangriming@scau.edu.cn (R.H.); zhqianw@scau.edu.cn (Q.Z.)

**Keywords:** green extraction, sulfated polysaccharides, density functional theory, molecular dynamics simulation, structure–activity relationship

## Abstract

Traditional polysaccharide extraction suffers from low efficiency and high energy consumption, while deep eutectic solvents (DESs) are promising sustainable solvents. This study used DES ChCl-LA (1:2) with ultrasonic assistance to extract polysaccharides from red alga *A.* *taxiformis*. Optimized via single-factor experiments and response surface methodology (350 W, 1:30 g/mL, 75 °C), the yield reached 11.28% ± 0.50% (1.5 times higher than that obtained by water extraction). Structural characterization revealed that the DES extract was an acidic polysaccharide, mainly composed of galactose (89.2%), glucose (4.9%), xylose (4.9%), and glucuronic acid (1.0%), with a weight-average molecular weight of 99.88 kDa. Density functional theory and molecular dynamics simulations showed that ChCl-LA enhanced galactose solubility via stronger hydrogen bonding (−25.33 vs. −5.06 kcal/mol for water). Notably, the immunological activity of the DES-extracted polysaccharide was significantly compromised compared to the water-extracted counterpart (*p* < 0.05). At a concentration of 0.25 mg/mL, the water-extracted polysaccharide-treated group exhibited a 33.98% higher neutral red phagocytosis rate in macrophages, a nitric oxide (NO) secretion level of 34.14 μmol/L (94.98% higher) compared with the DES-extracted polysaccharide group, as well as significantly higher secretion levels of tumor necrosis factor-α (TNF-α) and interleukin-6 (IL-6). The observed disparity in bioactivity is likely due to the distinct chemical profiles resulting from the two extraction methods, including the significantly reduced molecular weight and potential alterations of sulfation degree, monosaccharide composition, and protein content in the DES-extracted polysaccharide. This mechanistic perspective is supported by the relevant literature on the structure–activity relationships of polysaccharides. This study demonstrates the potential of ChCl-LA and elucidates the complex effects of extraction methods on polysaccharide’s structure and function, thereby informing the high-value utilization of *A. taxiformis* in functional foods.

## 1. Introduction

As integral components of marine ecosystems and critical reservoirs of natural products, seaweeds are rich in bioactive substances with significant application potential in biotechnology, pharmacology, and other fields, thus emerging as a global research focus [1]. Among diverse seaweed species, *Asparagopsis taxiformis* (commonly known as haicai or asparagus algae) has attracted substantial attention due to its unique ecological distribution (growing on coral reefs or coral substrates in subtidal zones) and excellent biological activities [2]. Studies have confirmed that when used as a feed additive, *A. taxiformis* can triple the immune response of farmed fish, significantly enhancing humoral immunity and innate cellular immune function [3]. Adding *A. taxiformis* containing 5% organic matter to ruminant feed reduces intestinal methane emissions by 99%, demonstrating remarkable potential for environmental regulation [4]. Further research has indicated that these activities are closely associated with its bioactive constituents, such as bromoform, phlorotannins, and polysaccharides [5]. However, existing studies on *A. taxiformis* have primarily focused on taxonomy and basic component analysis [6]. The extraction processes, structural characteristics, and functional activities of polysaccharides—the core bioactive substances—have not yet been systematically explored.

Currently, global challenges such as the high incidence of chronic diseases, escalating antibiotic resistance, and environmental degradation have underscored the importance of developing natural bioactive substances [7]. In particular, the global spread of coronavirus disease 2019 (COVID-19) has further highlighted the value of researching natural immunomodulators [8]. As natural immunomodulators, marine red algal polysaccharides—rich in sulfated groups—exhibit anti-inflammatory, antioxidant, and immunomodulatory activities, making them highly valuable in anti-tumor, antibacterial, and functional food development [9]. Notably, the biological activity of polysaccharides is closely linked to their structural characteristics (e.g., molecular weight, monosaccharide composition, and glycosidic bond types), while extraction methods are a key factor influencing polysaccharide structure and activity [10]. Although the traditional hot-water extraction method is widely used for its simplicity and low cost, it has inherent drawbacks stemming from its mechanism—relying on polysaccharide hydrophilicity to disrupt cell walls—resulting in long extraction times, high energy consumption, and low efficiency, which cannot meet the requirements for efficient polysaccharide preparation [11].

Deep eutectic solvents (DESs), as novel green solvents composed of hydrogen bond donors (HBDs) and hydrogen bond acceptors (HBAs), can form strong interactions with polysaccharides via intermolecular hydrogen bonds, electrostatic forces, and other interactions, thereby significantly improving extraction efficiency. Their solubility far exceeds that of conventional solvents [12]. For example, Wang et al. [13] found that the extraction yield of Cordyceps militaris polysaccharides using a choline chloride-succinic acid DES (12.78%) was significantly higher than that obtained via hot-water extraction (8.67%). Shafie, Yusof, and Gan [14] demonstrated that the yield of Averrhoa carambola fruit polysaccharides extracted with a choline chloride-citric acid monohydrate DES (14.44%) was markedly higher than that using a traditional citric acid solvent (9.34%). Feng et al. [15] reported that the extraction yield of Camellia oleifera polysaccharides with a choline chloride-propionic acid DES (14.2%) exceeded that from hot-water extraction (10.3%). Additionally, Wang et al. [16] compared the differences in biological activities of polysaccharides extracted from Tartary buckwheat sprouts using the DES method and the HWE method, and found that the secretion levels of NO, IL-6 and TNF-α in the DES-extracted polysaccharide group were all higher than those in the HWE-extracted polysaccharide group. This is attributed to the higher uronic acid content and lower degree of esterification (DE) of the polysaccharides extracted by DES.

These studies confirm the significant advantages of DESs in polysaccharide extraction. Notably, most previous studies took DES screening and extraction process optimization as their core objectives, focusing on improving extraction yield while lacking in-depth investigation into the extraction mechanism. Moreover, research on the mechanism of action of DESs has been limited to macroscopic explanations of solvent polarity affecting solubility [17], with insufficient understanding of solvent-solute intermolecular interactions and dissolution behavior at the microscopic level. Only by elucidating these intermolecular interactions can we tailor DES compositions to match the structural characteristics of target polysaccharides, thereby enhancing extraction specificity, optimizing polysaccharide bioactivity, and reducing the trial-and-error costs of DES screening [18]. In contrast, this study will focus on the investigation of polysaccharide extraction yield, extraction mechanism and immunomodulatory activity, using *A. taxiformis*—a marine red alga with great ecological and industrial potential—as the research object.

To address these research gaps and technical bottlenecks, this study focuses on the efficient development of *A. taxiformis* polysaccharides and undertakes the following work: preparing 12 types of DESs and screening the optimal extractant; optimizing extraction parameters via single-factor experiments combined with response surface methodology (RSM); characterizing polysaccharide structures using high-performance liquid chromatography (HPLC) and ion chromatography (IC); revealing the molecular mechanism underlying the efficient extraction of polysaccharides by DESs through a combination of density functional theory (DFT) and molecular dynamics (MD) simulations; and comparing the immunological activities of polysaccharides extracted by DES and hot-water methods using the RAW264.7 macrophage model. This study provides a theoretical basis for the efficient preparation of *A. taxiformis* polysaccharides and further lays a foundation for their potential application in functional food development.

## 2. Materials and Methods

### 2.1. Materials and Reagents

The red alga *A. taxiformis* used in this study were manually collected from the intertidal zone of the coastal waters of Naozhou Island, Zhanjiang, China, in May 2024. After being thoroughly rinsed with seawater and manually sorted to remove epiphytic algae, sediment, and other impurities, the samples were air-dried naturally, ground into powder, and passed through a 120-mesh standard sieve (aperture size: ca. 125 μm). The resulting powder was used for subsequent polysaccharide extraction. Cell counting kit-8 (CCK-8: C0048S), lipopolysaccharide (LPS: S1732-0.5 mg) assay kit, and nitric oxide (NO: S0021S) assay kit were purchased from Beyotime Biotechnology (Shanghai, China). All other reagents were of analytical purity.

### 2.2. Preparation of Deep Eutectic Solvents

**Figure 1:**
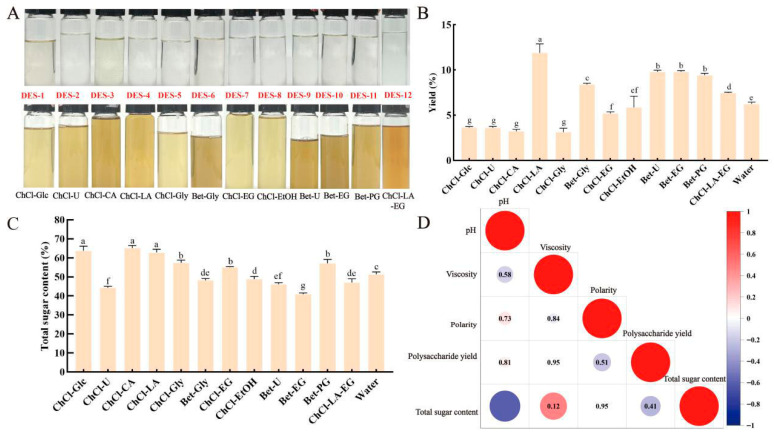
(**A**) Twelve prepared DESs (solvents) and extracts from different extraction solvents. (**B**) Effects of different DES types on the yield of *A. taxiformis* polysaccharides. (**C**) Effects of different DES types on total sugar content. (**D**) Correlations between physicochemical properties of different DESs and polysaccharide yield, as well as total sugar content. Values are expressed as mean ± SD (n = 3). Different letters indicate a different significance among groups at *p* < 0.05.

Referring to the method described by Zhu et al. (2025) [19], 12 different DESs were prepared. Briefly, the mixture prepared according to Supplementary Table S1 was added into a flask equipped with a stirring device, and then heated with stirring at 80 °C for 1.5 h until a stable and transparent liquid was formed in the flask (Figure 1A). Subsequently, a consistent 30% (*w*/*w*) water was added to all the DES mixtures while they were still hot, and mixed uniformly to reduce the viscosity of the solvents, thereby facilitating the extraction operation.

### 2.3. Determination of Physicochemical Properties of DESs

To investigate the effects of the physicochemical properties of the 12 types of DESs on polysaccharide extraction yield, this study determined the key physicochemical parameters of the DESs (with a mass fraction of 70%): their viscosities were measured using a rotational viscometer (Brookfield Engineering Laboratories, Inc., Middleboro, MA, USA); their pH values were measured directly in 70% DES–water mixtures with a pH meter, with the temperature set at 25 °C; and their polarities were analyzed via the Nile red dye probe method [20].

### 2.4. Extraction and Physicochemical Property Analysis of A. taxiformis Polysaccharides in the DES System

A sample of 2.0 g (dry weight) of *A. taxiformis* algal powder was weighed, and each of the 12 types of deep eutectic solvents (DESs) was added at a solid-to-liquid ratio of 1:30 (g/mL). The mixture was subjected to ultrasonic treatment in an ultrasonic cleaner (KQ-500DB, Kunshan Ultrasonic Instrument Co., Ltd., Kunshan, China) with a nominal power of 350 W and a fixed frequency of 40 kHz for 50 min; the temperature was controlled at 30 ± 2 °C during sonication via the instrument’s built-in temperature control function. Subsequently, the mixture was transferred to a digital constant-temperature water bath (HH-8, Jintan Medical Instrument Factory, Changzhou, China) for heating at 85 °C for 3.5 h with magnetic stirring at 300 rpm. After heating, the mixture was centrifuged at 8000 r/min for 15 min, and the supernatant was collected. Three volumes of absolute ethanol were added to the supernatant to achieve a final ethanol concentration of approximately 75% (*v*/*v*), which was then allowed to stand at 4 °C overnight. After centrifugation at 8000 r/min for 15 min again, the polysaccharide precipitate was obtained. The precipitate was washed with absolute ethanol three times and then dissolved in ultrapure water to form a polysaccharide solution. The solution was subjected to dialysis using a dialysis bag with a molecular weight cut-off (MWCO) of 3500 Da for 48 h (ultrapure water was changed every 6 h during dialysis) and subsequent freeze-drying to obtain the polysaccharides of *A. taxiformis*.

To analyze the physicochemical properties of the extracted polysaccharides, the following indices were determined: total polysaccharide content via the phenol-sulfuric acid method (using D-galactose as the standard), protein content using the BCA protein quantification method (with bovine serum albumin (BSA) as the standard), and sulfate group content through the barium chloride-gelatin turbidimetric method (employing potassium sulfate (K_2_SO_4_) as the standard). All determination results were expressed on a dry polysaccharide basis.

### 2.5. Optimization of Extraction Process for Polysaccharides from A. taxiformis

According to the preparation method described in Section 2.2, the DES that was identified to have the highest extraction yield was selected as the extraction solvent. The effects of key parameters on the extraction efficiency of polysaccharides from *A. taxiformis* were investigated, including solid-to-liquid ratios (1:20, 1:25, 1:30, 1:35, 1:40 g/mL), extraction temperatures (55 °C, 65 °C, 75 °C, 85 °C, 95 °C), and ultrasonic powers (250, 300, 350, 400, 450 W). Based on the single-factor experiments, a response surface experimental design was conducted. Herein, solid-to-liquid ratio (A), extraction temperature (B), and ultrasonic power (C) were set as independent variables, while the extraction yield of *A. taxiformis* polysaccharides was used as the response value. The factor levels of the response surface design are shown in Supplementary Table S2.

### 2.6. Molecular Weight and Monosaccharide Composition of Polysaccharides from A. taxiformis

After the polysaccharide solution was collected, the protein was removed using the Sevage method [21]. Prior to HPGPC analysis, dextran standards (with a molecular weight range of 6.3 kDa to 1000 kDa) were prepared as standard solutions: 5 mg of each dextran standard was accurately weighed, dissolved in 1 mL of the mobile phase, and formulated into a 5 mg/mL solution, which was then transferred to 1.8 mL sample vials for subsequent use. The HPGPC analysis was carried out on a Thermo U3000 system (Thermo Fisher Scientific, Waltham, MA, USA) equipped with an RID-20A refractive index detector (Shimadzu, Kyoto, Japan). Tandem BRT 105-103-101 gel columns (8 × 300 mm, Borui Saccharide, Wuxi, China) were adopted. The mobile phase was 0.5 M sodium chloride (NaCl) solution with an isocratic flow rate of 0.7 mL/min. The column temperature was maintained at 40 °C, and the injection volume was 100 μL. A calibration curve was constructed using the above-prepared dextran standard solutions for molecular weight calculation.

For monosaccharide composition analysis, 16 monosaccharide standards and the target samples (5 mg each) were accurately weighed and separately placed in ampoules, each added with 2 mL of 3 M TFA followed by hydrolysis at 120 °C for 3 h; the acid-hydrolyzed solutions were then precisely transferred to centrifuge tubes and dried by nitrogen blowing to completely remove TFA, after which the residues were dissolved in 5 mL of water and vortexed thoroughly to obtain standard stock solutions, while for sample pretreatment, 50 μL of the sample solution was pipetted and diluted with 950 μL of deionized water, centrifuged at 12,000 rpm for 5 min, and the supernatant was collected for subsequent analysis using a Dionex CarboPac PA20 column (Thermo Fisher Scientific, Sunnyvale, CA, USA; 3 × 150 mm) with mobile phases consisting of Phase A (ultrapure water), Phase B (15 mM NaOH), and Phase C (15 mM NaOH containing 100 mM NaOAc), at a flow rate of 0.3 mL/min, injection volume of 5 μL, and column temperature of 30 °C, with the elution gradient (time vs. phase volume ratio, V/V) set as follows: 0–18 min (98.8:1.2:0), 18–20 min (linear gradient from 98.8:1.2:0 to 50:50:0), 20–30 min (50:50:0), 30.1–46 min (0:0:100), 46.1–50 min (0:100:0), and 50.1–80 min (98.8:1.2:0), and an electrochemical detector was used for detection.

### 2.7. Density Functional Theory Analysis

Density functional theory (DFT) calculations were performed in the gas phase using Gaussian 09 Revision D.01 software (Gaussian Inc., Wallingford, CT, USA), following the method described by Liu [22]. Explicit solvation was not incorporated in the calculations, except for the use of cluster models. The initial structures of the key molecules were obtained from the PubChem database, including galactose (PubChem CID: 6036), choline chloride (CID: 6209), lactic acid (CID: 612), and water molecule (CID: 962). The calculation procedure was carried out as follows: First, the structures of these four individual molecules were geometrically optimized, and differences in their Mulliken atomic charges and molecular electrostatic potentials were analyzed. Subsequently, the binding energy between DES and galactose was calculated; it should be noted that the DES component in the DES–galactose complex was composed of choline chloride (ChCl) and lactic acid (LA) at a molar ratio of 1:2, rather than a larger cluster structure. The number and bond lengths of hydrogen bonds during their interaction were optimized to determine the most stable geometric configuration. In parallel, the binding energy, number of hydrogen bonds, and bond lengths between water molecules and galactose were calculated using the same method, in order to reveal the molecular mechanism underlying the efficient extraction of *A. taxiformis* polysaccharides by DES at the molecular level. The binding energy of the interaction between galactose and DES was calculated using the following full explicit equation: Ebinding = EDES-Galactose−(EDES + EGalactose)Ebinding = EDES-Galactose−(EDES + EGalactose), where EDES-Galactose represents the energy of the DES-galactose complex; EDES represents the energy of the isolated DES molecule; EGalactose represents the energy of galactose.

### 2.8. Molecular Dynamics Simulation

Molecular dynamics (MD) simulations were performed using AMBER 22 software [23]. The structures of key molecules were retrieved from the PubChem database (galactose CID: 6036; choline chloride (ChCl) CID: 6209; lactic acid (LA) CID: 612; water molecule CID: 962). Two systems were constructed via Packmol software (Version 20.15.1) in cubic boxes (60.0 Å × 60.0 Å × 90.0 Å): ① galactose-ChCl-LA system (50 ChCl, 100 LA, and 50 galactose molecules); ② galactose-water system. Molecules were uniformly distributed to avoid spatial overlap, and the initial structures were output as PDB-format files for subsequent MD simulations. Prior to simulations, atomic charges of small molecules were derived via the Antechamber module of AMBER combined with the HF SCF/6-31G method in Gaussian 09. Force field settings: GAFF2 for small molecules, ff14SB for proteins (if applicable), and TIP3P water model for the water system. Hydrogen atoms were added and the systems were solvated in truncated octahedral TIP3P solvent boxes using the LEaP module of AMBER 22, followed by generation of topology and parameter files. Simulation procedure: energy minimization (2500 steps of steepest descent + 2500 steps of conjugate gradient) → temperature ramp (0 K to 298.15 K over 200 ps at constant volume) → 500 ps NVT equilibration → 500 ps NPT equilibration → 200 ns NPT production simulation under periodic boundary conditions. Simulation parameters: non-bonded cutoff distance = 10 Å; long-range electrostatic interactions calculated via the Particle Mesh Ewald (PME) method; SHAKE method used to constrain hydrogen atom bond lengths; Langevin algorithm for temperature control (collision frequency γ = 2 ps^−1^); system pressure = 1 atm; integration time step = 2 fs; trajectories saved every 10 ps for analysis. The concentrations of constructed systems were consistent with realistic experimental conditions.

### 2.9. Immunostimulatory Effects of Crude Polysaccharides from A. taxiformis on RAW 264.7 Macrophages

#### 2.9.1. Cell Culture

Cell culture was performed as described by Sun [24]. RAW 264.7 cells were seeded in complete Dulbecco’s modified Eagle’s medium (DMEM) supplemented with 10% fetal bovine serum (FBS) and 1% penicillin–streptomycin (P/S). The cells were cultured in a humidified cell incubator at 37 °C with 5% CO_2_ and 95% relative humidity. Subculturing was performed every 24 h when the cells reached 80% confluence in the culture flask, and cells in the logarithmic growth phase were used for subsequent experiments.

#### 2.9.2. Determination of Cell Viability

The One-step Chromogenic LAL Endotoxin Assay Kit (Beyotime Biotechnology, Shanghai, China) was used to determine the content of LPS in polysaccharides extracted from *A. taxiformis*. The specific operation was implemented according to the descriptions provided within the Kit. RAW 264.7 cells at a density of 1 × 10^5^ cells/mL were seeded in a 96-well plate at 100 μL/well. After incubation in the cell incubator (37 °C, 5% CO_2_) for 24 h, the medium was discarded. Subsequently, 100 μL of medium containing *A. taxiformis* sulfated polysaccharides (ATSP) at different concentrations (0.25–1 mg/mL) was added to each well. The medium without ATSP was used as the control group. Four replicate wells were prepared for each group. After 24 h of incubation in the cell incubator (37 °C, 5% CO_2_), 10 μL of CCK-8 solution was added to each well. Following another incubation at 37 °C for 1 h, the absorbance at 450 nm was measured with a microplate reader, and cell viability was calculated accordingly.

#### 2.9.3. Determination of Cell Phagocytic Activity

After 24 h of cell culture, the culture supernatant was discarded. Cells were treated according to the method described in Section 2.9.2, with control, sample, and positive control (1 μg/mL LPS) groups set up. After 24 h of reaction, 100 μL of 0.1% (*w*/*v*) neutral red staining solution was added to each well. Following incubation at 37 °C for 30 min, the cells were washed three times with phosphate-buffered saline (PBS). Subsequently, 200 μL of lysis buffer (1:1 *v*/*v* anhydrous ethanol-glacial acetic acid mixture) was added to each well, and the cells were lysed at room temperature for 30 min. The absorbance at 540 nm was measured using a microplate reader (Varioskan LUX Multimode microplate reader, Thermo Fisher Scientific, Waltham, MA, USA). The phagocytic activity was calculated as the neutral red phagocytosis rate relative to the control group.

#### 2.9.4. Determination of Cytokines NO, TNF-α and IL-6

The levels of NO, TNF-α, and IL-6 released by RAW 264.7 cells were measured using the Griess assay (for NO) and enzyme-linked immunosorbent assay (ELISA, for TNF-α and IL-6). After 24 h of cell culture (following the same cell treatment procedure as described in Section 2.9.3), 50 µL of standards and samples were added to each well of a 96-well plate, respectively. The secretion level of NO in the supernatant was determined according to the manufacturer’s instructions of the Griess assay kit, the standard curve equation was y = 0.0077x + 0.0473, with a coefficient of determination of R^2^ =0.9992. The levels of TNF-α and IL-6 in the supernatant were detected following the operating protocols of their corresponding ELISA kits.

### 2.10. Statistical Analysis

All experiments were repeated three times, and the data are presented as mean ± standard deviation (Mean ± SD). The results were analyzed using one-way analysis of variance (one-way ANOVA) in SPSS 27 software, followed by Duncan’s multiple range test. The significance level (α) was set at 0.05. Additionally, GraphPad Prism 8.3.0 software and Design-Expert 13.0 was used for graphing.

## 3. Results and Discussion

### 3.1. Extraction Efficiency Influenced by DESs and Their Relation with Physicochemical Properties

To screen the most effective DES, a total of 12 types of DESs with 30% water content were prepared for the extraction of crude polysaccharides from *A. taxiformis* (Figure 1A), with polysaccharide yield and total sugar content set as the key indicators for screening. Compared with the hot water extraction method (Figure 1B), 6 types of DESs significantly increased the polysaccharide yield (*p* < 0.05), namely ChCl-LA (choline chloride-lactic acid), Bet-Gly (betaine-glycerol), Bet-U (betaine-urea), Bet-EG (betaine-ethylene glycol), Bet-PG (betaine-propylene glycol), and ChCl-LA-EG (choline chloride-lactic acid-ethylene glycol). Notably, the polysaccharide yield of ChCl-LA reached 11.88% ± 1.00%, which was 1.9 times that of hot water extraction (6.23% ± 0.21%). In addition, the total sugar content of extracts obtained using 6 types of DESs was significantly higher than that of the aqueous solution (hot water extraction system). These DESs were ChCl-Glc (choline chloride-glucose), ChCl-CA (choline chloride-citric acid), ChCl-LA, ChCl-Gly (choline chloride-glycerol), ChCl-EG (choline chloride-ethylene glycol), and Bet-PG. Among them, ChCl-CA had the highest total sugar content of 65.18% ± 1.29% (Figure 1C). Therefore, based on the results of polysaccharide yield and total sugar content, ChCl-LA with 30% water content (total sugar content: 62.83% ± 1.73%) was selected as the solvent for subsequent extraction.

DESs are composed of HBDs and HBAs. They can form hydrogen bonds between DES molecules and polysaccharide compounds, thereby enhancing the solubility of polysaccharides in the solvent. However, the solubility of polysaccharides is influenced by the physical properties of DESs, such as viscosity, polarity, and pH [25]. According to the cavity theory, high viscosity increases steric hindrance in DESs. After appropriate dilution with water, the viscosity of the solvent decreases, which facilitates enhanced mass transfer of solutes into the solvent and thus promotes polysaccharide dissolution. Conversely, excessive water molecules disrupt the hydrogen bond network, which is detrimental to the extraction process [26]. Figure 1D illustrates the correlations between the physicochemical properties of 12 DESs and polysaccharide yield as well as total sugar content. In this study, we found that polysaccharide yield is positively correlated with pH (r = 0.81), negatively correlated with polarity (r = −0.51), and exhibits an extremely weak correlation with viscosity (indicated by the nearly white color in the plot). Additionally, total sugar content is negatively correlated with pH (r = −0.8, shown as dark blue), while its correlations with viscosity (r = 0.12) and polarity are relatively weak (indicated by the nearly white color). These results indicate that the pH of the solvent is a core factor affecting both polysaccharide yield and total sugar content: an appropriate pH can not only enhance polysaccharide yield through a weakly alkaline environment but also prevent a decrease in total sugar content caused by excessively high pH; in contrast, overly acidic or alkaline conditions may induce polysaccharide degradation (e.g., glycosidic bond cleavage) or elution of impurities, which not only reduce the efficiency of polysaccharide extraction but also dilute the proportion of total sugar [27].

### 3.2. Single-Factor Experiments for Synergistic Ultrasonic-Assisted Extraction of Polysaccharides from A. taxiformis Using DESs

**Figure 2:**
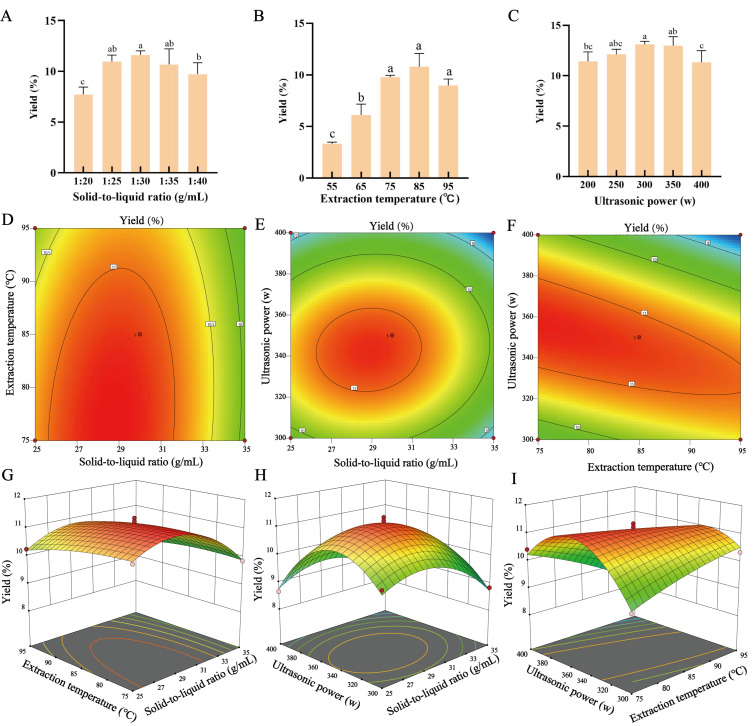
Single-factor experiments and response surface methodology for optimization of polysaccharide extraction from *A. taxiformis*. (**A**–**C**) Effects of solid-to-liquid ratio, extraction temperature, and ultrasonic power on crude polysaccharide yield. (**D**,**G**) Interaction between solid-to-liquid ratio and extraction temperature. (**E**,**H**) Interaction between solid-to-liquid ratio and ultrasonic power. (**F**,**I**) Interaction between extraction temperature and ultrasonic power. Values are expressed as mean ± SD (n = 3). Different letters indicate a different significance among groups at *p *< 0.05.

To evaluate the effects of individual factors on polysaccharide extraction based on ChCl-LA and determine the optimal range of each parameter, single-factor experiments were conducted. As shown in Figure 2A, the polysaccharide yield reached the maximum when the solid-to-liquid ratio was 1:30 (g/mL) with other parameters set as follows: heating time of 3.5 h, extraction temperature of 85 °C, and ultrasonic power of 350 W. This phenomenon indicates that an appropriate increase solid-to-liquid ratio helps increase the relative contact area between the solvent and the sample, thereby enhancing the solubility of polysaccharides in the solvent [28]. However, when the solid-to-liquid ratio exceeds 1:30, the contribution of this factor to polysaccharide yield diminishes. Similarly, the maximum polysaccharide yield was achieved at an extraction temperature of 85 °C, with other parameters fixed at 3.5 h, a solid-to-liquid ratio of 1:30 (g/mL), and an ultrasonic power of 350 W. This is because increased temperature can enhance the fluidity of the extraction solution, reduce solvent viscosity and surface tension, thereby promoting extraction [29]; however, when the temperature exceeds 85 °C, polysaccharide degradation caused by thermal effects offsets the improvement in mass transfer rate induced by high temperature [30]. Furthermore, the highest polysaccharide yield was obtained at an ultrasonic power of 300 W, with other parameters fixed at a heating time of 3.5 h, a solid-to-liquid ratio of 1:30 (g/mL), and an extraction temperature of 85 °C. This is because as ultrasonic power increases, cavitation bubbles collapse more violently, generating stronger shear forces to promote solute dissolution. Nevertheless, excessively high power leads to intense sonochemical effects caused by cavitation bubble rupture, resulting in polysaccharide degradation and a subsequent decrease in yield [31].

### 3.3. RSM Optimization of ChCl-LA-UAE on Yield of A. taxiformis Sulfated Polysaccharides

To optimize the process parameters, it is necessary to identify the key factors that significantly affect the process. Preliminary single-factor experiments showed that extraction temperature (75 °C, 85 °C, 95 °C), ultrasonic power (300, 350, 400 W), and solid-to-liquid ratio (1:25, 1:30, 1:35 g/mL) were the three core variables for the extraction of polysaccharides using DESs (Figure 2). Based on the results of the single-factor experiments, a Box–Behnken (BB) design was employed to investigate the interaction effects among these three variables. The experimental design of RSM and the corresponding polysaccharide yield results are presented in Supplementary Table S3, while the analysis of variance for the significance of the regression model coefficients is listed in Table 1.

From Table 1, the quadratic regression model for the polysaccharide yield of *A. taxiformis* with respect to the solid-to-liquid ratio (A, g/mL), extraction temperature (B, °C), and ultrasonic power (C, W) is as follows:

$$Y=11.16-0.3825\;A\;-\;0.22\;B\;-\;0.365\;C\;-\;0.77\;\mathrm{BC}\;-\;0.8695{\;A}^{2}\;-\;1.42{\;C}^{2}$$

Notably, the quadratic terms (A^2^, C^2^) in the equation have negative coefficients (−0.8695 for A^2^, −1.42 for C^2^). This indicates that the response surface curves corresponding to liquid-to-solid ratio and ultrasonic power present a convex shape, a key feature confirming the existence of a maximum yield point, which provides theoretical support for identifying the optimal extraction conditions. The model exhibited a *p*-value < 0.0001, a coefficient of determination (R^2^ = 0.9868), and an adequate precision of 20.7203, indicating high fitting degree and reliability of the equation. The order of the influence of the factors on the polysaccharide extraction rate was as follows: solid-to-liquid ratio (F = 34.69) > ultrasonic power (F = 31.59) > extraction temperature (F = 11.48). The steepness of the response surface reflects the interaction effects between factors and their relative significance on the polysaccharide extraction rate of *A. taxiformis*. As shown in Figure 2E–H, the response surface curve for the interaction between solid-to-liquid ratio (A) and ultrasonic power (C) was the steepest, indicating that this interaction had the most significant effect on the polysaccharide extraction rate of *A. taxiformis*. Similarly, the contour plots also confirmed the most prominent interaction between solid-to-liquid ratio (A) and ultrasonic power (C). These interactions enhanced the solubility and stability of polysaccharides during extraction, thereby increasing the polysaccharide yield [32].

Through RSM analysis, the optimal extraction conditions for maximum polysaccharide yield were predicted as follows: solid-to-liquid ratio of 1:28.648 (g/mL), extraction temperature of 75 °C, and ultrasonic power of 356.504 W. Under these conditions, the predicted yield of *A. taxiformis* sulfated polysaccharides (ATSP) was 11.33%, while the actual verified value was 11.28% ± 0.50%, which was 1.5 times higher than that of water-extracted polysaccharides under the same conditions (7.68% ± 0.58%). Additionally, the close agreement between the model predictions and actual values demonstrated the accuracy and robustness of the RSM model. Considering practical operability, the optimal process was adjusted to: solid-to-liquid ratio of 1:30, extraction temperature of 75 °C, and ultrasonic power of 350 W. Substitution of these practical conditions into the regression equation yielded a predicted yield of 11.38%, corresponding to a negligible yield loss of only 0.05% compared with the theoretical optimum (11.33%). It would be helpful to explicitly note that single-factor optima can differ from multivariate optima due to interaction effects and the specific response (crude vs. sulfated polysaccharide yield).

### 3.4. Physicochemical Properties and Structural Characterization of Polysaccharides from A. Taxiformis

This study compared the effects of DES extraction and hot water extraction on the physicochemical properties and structural characteristics of polysaccharides. The results show that the contents of total sugar, protein, and sulfate groups in the polysaccharides extracted by ChCl-LA (DES) were 62.5% ± 1.25%, 4.95% ± 0.07%, and 14.17% ± 2.75%, respectively. Under the same extraction conditions, the contents of total sugar, protein, and sulfate groups in the polysaccharides extracted by hot water were 51.38% ± 1.22%, 10.10% ± 0.18%, and 15.53% ± 0.75%, respectively. These results indicated that there were significant differences in selectivity for components between ChCl-LA (DES) extraction and hot water extraction for the extraction of polysaccharides from *A. taxiformis*.

**Figure 3:**
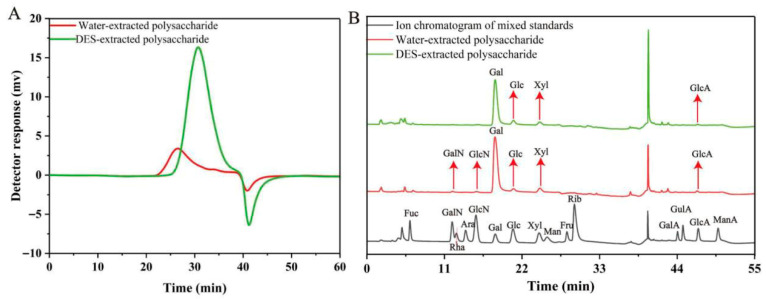
Molecular weight (**A**) and monosaccharide composition (**B**) of polysaccharides from *A. taxiformis*.

Furthermore, high-performance liquid chromatography (HPLC) and ion chromatography (IC) were used to analyze the differences in molecular weight and monosaccharide composition between the two polysaccharide samples. The results (Figure 3A) show that the weight-average molecular weight (Mw) of the hot water-extracted polysaccharides was 812.53 kDa, with a polydispersity index of 1.017; in contrast, the Mw of the DES-extracted polysaccharides was 99.88 kDa, with a polydispersity index (PDI) of 1.027. The PDI is a key parameter characterizing the uniformity of polysaccharide molecular weight distribution: the smaller the ratio (Mw/Mn) (closer to 1), the more uniform the molecular weight distribution of the sample [33]. This suggested that the molecular weight distributions of the polysaccharides obtained by both hot water extraction and DES extraction were relatively uniform. Notably, the Mw of the hot water-extracted polysaccharides was 8.13 times that of the DES-extracted ones, suggesting that DES extraction is more conducive to the acquisition of low-molecular-weight polysaccharides. Feng et al. extracted polysaccharides from oil-tea camellia husks using DES (ChCl-LA), and found that the molecular weight of polysaccharides treated with acidic DES was significantly lower than that obtained by traditional hot water extraction, and the antioxidant activity was higher [15]. This is consistent with the conclusion of this study, indicating that the acidic environment of DES may reduce the molecular weight of polysaccharides through the selective hydrolysis of glycosidic bonds.

Moreover, the results of monosaccharide composition (Figure 3B) show that the hot water-extracted polysaccharides were mainly composed of galactose (90.2%), glucose (3.2%), xylose (4.8%), glucuronic acid (1.1%), galactosamine hydrochloride (0.3%), and glucosamine hydrochloride (0.3%), whereas the polysaccharides obtained by DES extraction mainly consisted of galactose (89.2%), glucose (4.9%), xylose (4.9%), and glucuronic acid (1.0%). These results reveal that the polysaccharides obtained by both extraction methods are acidic polysaccharides (due to the presence of glucuronic acid and sulfate groups), and the proportion of galactose in both exceeds 89%, suggesting that the extracted polysaccharides are mainly galactose sulfate. However, there are significant differences in monosaccharide composition and molar percentage between the polysaccharides extracted by the two methods, which may be closely related to differences in their biological activities. Such differences (e.g., reduced sulfate content, eliminated amino sugars, and distinct Mw) may potentially affect immunoreceptor recognition by altering charge density and chain length, though further validation is required. Yi et al. found that the monosaccharide composition of polysaccharides varies due to multiple factors, such as the pH value of the extraction solvent, temperature, extraction method, and time [34]. Compared with traditional methods, the use of DES as an alternative solvent enables more abundant discoveries of structural characteristics, which indicates that its impact on polysaccharide structure has significant research value. It should be noted that the lack of glycosidic linkage pattern analysis (e.g., via NMR) in this study limits the depth of structure–activity relationship conclusions.

### 3.5. Molecular Mechanism of Polysaccharide Extracted Using DESs

DFT can predict the solubility of polysaccharides in DESs and evaluate the solubility relationship between polysaccharide structures and DES components by calculating parameters such as intermolecular interaction energy and charge distribution. Relevant studies have confirmed its effectiveness in analyzing the interaction mechanism between polysaccharides and DESs. For example, Guo et al. (2025) combined DFT calculations with MD simulations to elucidate that type II DESs disrupt and reconfigure the hydrogen bond network of galactomannans by providing a large number of anions and neutral hydrogen bonds [18], thereby achieving a significant physical dissolution effect. Cao et al. (2017) used DFT to study the dissolution mechanism of α-cyclodextrin and chitobiose in 1-ethyl-3-methylimidazolium acetate, confirming that the anions and cations of ionic liquids can promote dissolution through the synergistic effect of hydrogen bonds and van der Waals forces [35]. To reveal the intrinsic mechanism underlying the higher extraction efficiency of choline chloride-lactic acid (ChCl-LA) compared with water, DFT was employed in this study. Based on the fact that galactose accounts for 89.2% of the polysaccharides extracted by DESs and is the main monosaccharide component, galactose was selected as the solute. Meanwhile, ChCl-LA and water were chosen as solvents to compare the differences in their effects. Using the Gaussian 16 program, geometric optimization of galactose, ChCl-LA, and H_2_O molecules was performed at the B3LYP/6-31G(d,p) theoretical level (incorporating GD3BJ dispersion correction). The Mulliken Charge distribution and electrostatic potential surfaces were calculated to analyze the driving mechanism of intermolecular interactions from the perspective of charge.

**Figure 4:**
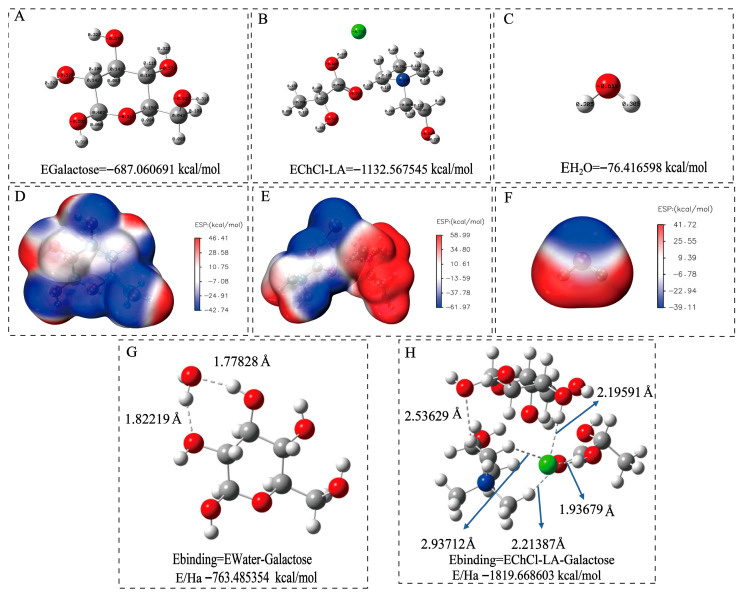
Optimized molecular structures with Mulliken Charges (**A**–**C**) and electrostatic potential (ESP) maps of Galactose, ChCl-LA (DES), and H_2_O (**D**–**F**) (red regions indicate positive electrostatic potential, while blue regions indicate negative electrostatic potential). (**G**) Molecular structure of water molecule bound to galactose. (**H**) Molecular structure of deep eutectic solvent (DES) bound to galactose.

As shown in Figure 4A–C, the optimized structures of galactose, ChCl-LA, and water molecules (H_2_O) are presented, respectively. The total energy (E/Ha, where Ha stands for Hartree) labeled below reflects the thermodynamic stability of the molecules. The total energy of ChCl-LA (−1132.57 Ha) is significantly more negative (indicating higher stability) than that of galactose (−687.06 Ha) and water (−76.42 Ha), which is attributed to the strong electrostatic interactions between choline cations (Ch^+^) and lactic acid anions (LA^−^) in the DES system, making its structure more stable. ESP maps reveal molecular surface charge distribution (red: positive potential; blue: negative potential) to predict key intermolecular interaction sites [36]. For galactose (Figure 4D–F), with an ESP range of −42.74 to 46.41 kcal/mol, hydroxyl oxygen atoms (negative potential) and hydrogen atoms (positive potential) serve as key interaction sites for forming intermolecular bonds. In contrast, ChCl-LA exhibits enhanced charge complementarity between Ch^+^ and LA^−^, providing abundant polar sites to interact with galactose—facilitating the formation of extensive hydrogen bonds and improving galactose solubility. Water also has positive/negative potential regions but lacks such abundant interaction sites.

The hydrogen atoms of water molecules form hydrogen bonds with the hydroxyl oxygen atoms of galactose in Figure 4G–H. Meanwhile, the oxygen atom of a water molecule forms a hydrogen bond with the hydroxyl hydrogen atom of another site on galactose, with a binding energy of −5.06 kcal/mol for this interaction. Likewise, the chlorine atom of choline chloride forms a hydrogen bond with the hydroxyl hydrogen of galactose, and the hydrogen atoms of choline chloride form hydrogen bonds with the hydroxyl oxygen atoms of galactose. Additionally, the chlorine atom of choline chloride forms a hydrogen bond with the hydrogen atom of lactic acid, and the hydrogen atoms of choline chloride form hydrogen bonds with the double-bond oxygen atoms of lactic acid; the binding energy for the interaction between ChCl-LA and galactose is −25.33 kcal/mol.

This indicates that the absolute binding energy between ChCl-LA and galactose is much higher (more negative) than that between water and galactose. Higher absolute binding energy (more negative) indicates stronger DES–galactose interactions and thus greater solubilizing power. This may be attributed to the formation of multiple hydrogen bonds (bond lengths: 2.536, 2.937, 2.214, 1.937, and 2.196 Å, respectively) between ChCl-LA and galactose after binding, which enhances the solubility of galactose (Figure 4H). In contrast, only two hydrogen bonds (bond lengths: 1.822 and 1.778 Å, respectively) are formed between water and galactose after binding (Figure 4G). Studies have shown that hydrogen bonds play a crucial role in the formation of DESs and their solvation processes. The enhanced extraction efficiency of polysaccharides using DESs can be attributed to the formation of hydrogen bonds between galactose molecules and DES molecules; this interaction enhances the solubility of galactose in DES systems [37]. In summary, data on binding energy, number of hydrogen bonds, and bond lengths indicate that the DES ChCl-LA exhibits much higher solubility for galactose than water, which is consistent with the experimental results in Figure 1B.

### 3.6. Dissolution Behavior Characteristics of Polysaccharides from A. taxiformis in ChCl-LA Solvent

Based on DFT calculations, MD simulations enable more intuitive observation of dynamic processes, such as how DESs disrupt the hydrogen bond networks of polysaccharides and bind to polysaccharide molecules, thereby further clarifying the polysaccharide extraction mechanism. For example, Feng et al. (2020) used MD simulations to study the diverse conformational properties of a single polysaccharide chain containing 12 glucose units in an aqueous solution, and explored the formation mechanism of these conformational properties from the microscopic perspective of intramolecular and intermolecular non-covalent interactions [38]. Liu et al. (2025) employed MD simulations to investigate the mechanism underlying the efficient extraction of raspberry polyphenols by DESs, finding that choline chloride-fructose (ChCl-Fru) significantly enhances the interactions between solutes and solvents, and exhibits particularly higher extraction efficiency for delphinidin-3-O-glucoside [39]. Notably, the cluster aggregation behavior observed in MD simulations does not directly correspond to macroscopic phase behavior; these simulations solely provide microscopic insights into intermolecular interaction strength and solvation patterns, rather than characterizing full-scale dissolution thermodynamics. Thus, this study used MD simulations to investigate the molecular mechanism behind the efficient extraction of polysaccharides from *A. taxiformis* by the DES ChCl-LA.

**Figure 5:**
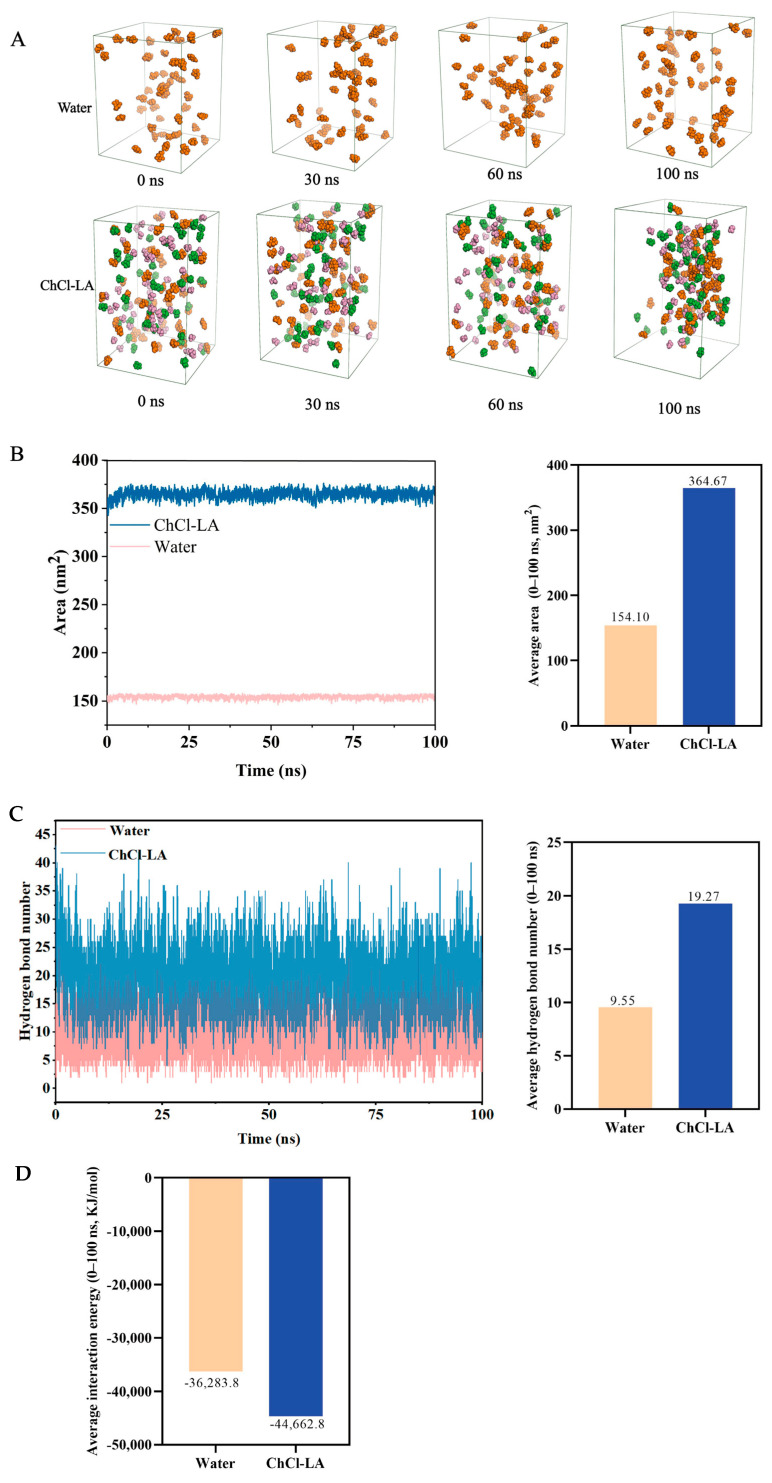
(**A**) Visual states of galactose molecules dissolved in different solvent systems during 0–100 ns molecular dynamics simulations. (**B**) Solvent-accessible surface area (SASA) and its average values. (**C**) Number of hydrogen bonds and its corresponding average values. (**D**) Variations in average binding energy of different solvents over 0–100 ns.

Figure 5A presents the molecular distribution characteristics of galactose in two solvent systems: water and ChCl-LA. For the dissolution state of galactose in the water system: within 0–30 ns, galactose molecules marked by orange spheres are scattered throughout the simulation box, with only scattered contact between molecules and no obvious clustered structures formed; when the simulation time is extended to 60–100 ns, the molecules still maintain a dispersed state overall without large-scale aggregation. This indicates that galactose exhibits good stability in water solvent and is not prone to spontaneously forming large aggregates. In contrast, the dissolution behavior of galactose in the ChCl-LA system exhibits significant differences. At 0–30 ns, galactose, lactic acid anions, choline cations, and chloride ions are uniformly dispersed, and some molecules have already formed small aggregates; during 60–100 ns, various molecules further approach each other and exist in the form of closely bound aggregates, forming multiple dense clusters. This phenomenon suggests that there is a strong mutual attraction between ChCl-LA and galactose during the simulation, and the interactions between components strengthen over time. Overall, under the same temperature and pressure, the strength of intermolecular interactions in the ChCl-LA system is significantly higher than that in the water solvent system, making it more likely to induce the formation of aggregated structures.

SASA is an important metric describing the contact degree between galactose molecules and solvent molecules in the simulated system, and can quantitatively reflect the dissolution and dispersion of galactose in various solvents [40]. The results in Figure 5B indicate that the SASA value of galactose in the water system is maintained at 154.10 nm^2^ overall, revealing that galactose molecules do not form large-scale clusters during the simulation, and that their surface area is relatively small and constant. In contrast, galactose in the ChCl-LA system undergoes rapid initial aggregation; after 10 ns, its SASA value jumps to 364.67 nm^2^, causing a large number of galactose molecules to be exposed to the solvent and significantly increasing the total SASA of the system. This difference in the contact degree between solute and solvent explains why ChCl-LA exhibits good solubility for galactose. When investigating the mechanism of glabridin extraction using a natural DES (ChCl-LA), Xing et al. also explored the influence of SASA and dispersion state on extraction efficiency, and obtained similar conclusions [41].

Figure 5C shows the variations in the number of hydrogen bonds formed between galactose and solvents in both water and ChCl-LA systems. Within 0–100 ns, the number of hydrogen bonds formed between galactose and ChCl-LA is significantly higher than that between galactose and water. The average number of hydrogen bonds formed between galactose and ChCl-LA is 19.27, which is twice that formed between galactose and water. The smaller number of hydrogen bonds in the water system reflects the limited binding capacity between components in a single-component solvent. In contrast, in the mixed solvent system, more diverse hydrogen bond networks are formed among galactose, lactic acid, choline cations, and chloride ions. This indicates that the continuous formation and breaking of hydrogen bond networks among these components is an important manifestation of the driving force for their aggregation, which is consistent with the conclusions from DFT calculations. Additionally, the binding energy results further clarify the molecular interactions between solute and solvent (Figure 5D). Galactose exhibits the lowest interaction energy in ChCl-LA (with an average interaction energy of −44,662.8 kJ/mol) and the highest interaction energy in water (with an average interaction energy of −36,283.8 kJ/mol).

These results suggest that the natural deep eutectic solvent (DES) choline chloride-lactic acid (ChCl-LA) exhibits a lower interaction energy with galactose, which facilitates the formation of a more stable system and thereby promotes the efficient extraction of galactose in the ChCl-LA system. This is consistent with the findings of Huang et al., who calculated intermolecular interaction energies via molecular dynamics (MD) simulations and demonstrated that, compared with ethanol, the acidified choline chloride-citric acid (ChCl-CA) DES system has a noncovalent interaction energy of −1329.74 kcal/mol, which is significantly lower than that of acidified ethanol (−715.02 kcal/mol) [42]. It should be noted that although MD simulations can intuitively reveal the microscopic molecular interaction mechanisms of the extraction process and provide robust support for the elucidation of polysaccharide extraction mechanisms, the MD simulations in this study still have several limitations that need to be briefly addressed: (1) The finite simulation box size and high nominal concentrations may deviate from actual experimental conditions and introduce boundary effects; (2) The calculated hydrogen bond numbers and interaction energies are subject to force-field-dependent uncertainties, which may affect the quantitative accuracy of the results.

### 3.7. Immune-Enhancing Effects of ATSP on RAW264.7 Macrophages

**Figure 6:**
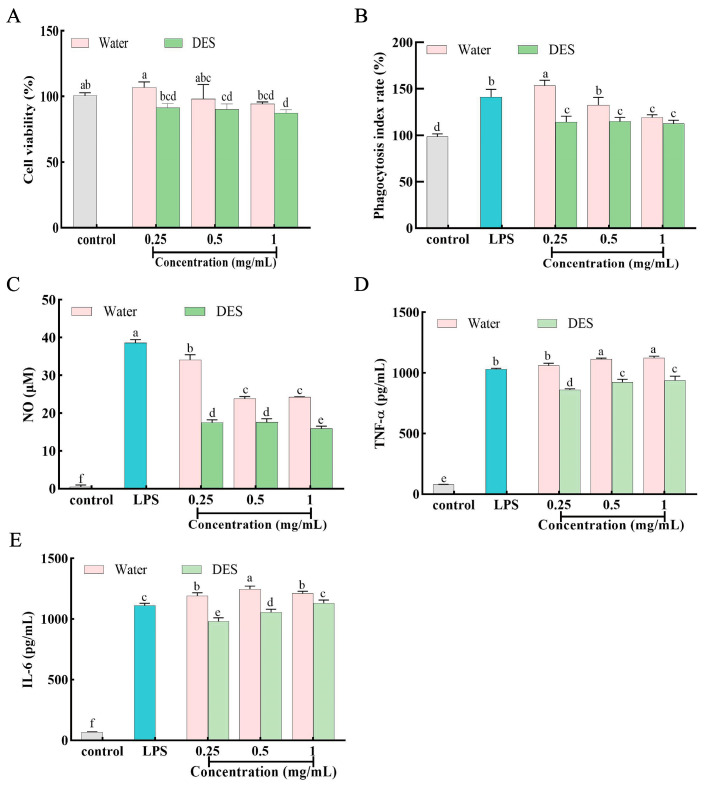
Immune-enhancing effects of ATSP on RAW264.7 macrophages. (**A**) Cell viability. (**B**) Phagocytic activity. (**C**) Secretion of NO. (**D**) TNF-α secretion. (**E**) IL-6 secretion. Values are expressed as mean ± SD (n = 3). Different letters indicate a different significance among groups at *p* < 0.05.

Lipopolysaccharide (LPS) contamination is inevitably present in natural extracts [43]. To eliminate the potential interference of LPS contamination on the experimental results, the One-step Chromogenic LAL Endotoxin Assay Kit was used to determine the content of LPS in polysaccharides extracted from *A. taxiformis*. The results of the LAL endotoxin assay showed that the polysaccharide solutions treated with LAL did not turn yellow, indicating that no endotoxin was detected in the polysaccharide samples at the concentration of 1 mg/mL and neither of the two polysaccharides was contaminated with LPS. Therefore, the interference of endotoxin can be excluded in the subsequent immunological experiments. As depicted in Figure 6A, within the concentration range of 0.25–1 mg/mL, the cell viability remained above 85%. This indicates that the polysaccharides extracted using the two solvents neither inhibit the proliferation of RAW 264.7 cells nor exert cytotoxic effects on macrophages. Meanwhile, when the concentration was 0.25 mg/mL, the polysaccharides extracted by hot water exhibited the highest macrophage viability, representing a 16.72% increase compared with those extracted by the DES method. Based on the cell viability assay results, the concentration range of 0.25–1 mg/mL was selected for subsequent experiments.

Phagocytosis of macrophages is a key process in the body’s innate immune response for resisting antigen invasion and clearing damaged cells [44]. After treatment with ATSP for 24 h, the phagocytic activity of RAW 264.7 macrophages was significantly enhanced (Figure 6B), indicating that polysaccharides extracted by both hot water and DES extraction methods can effectively promote the phagocytic function of macrophages (*p* < 0.05). Among them, when the concentration of polysaccharides extracted by the hot water method was 0.25 mg/mL, the neutral red phagocytosis rate reached the highest value, which was 33.98% higher than that of polysaccharides extracted by the DES method (*p* < 0.05). Studies have confirmed that the immunomodulatory activity of polysaccharides is closely related to their molecular weights. Polysaccharides with a molecular weight greater than 100 kDa usually exhibit higher activity, possibly because they have more binding sites for immune receptors, thereby enhancing interactions with receptors [45]. For example, Liu et al. (2022) found that the razor clam polysaccharide SCP-1-1 (molecular weight of 440.0 kDa, composed of glucose and mannose) could significantly improve the phagocytic capacity of RAW264.7 cells and promote the secretion of NO and cytokines such as TNF-α, IL-6, and IL-1β [46]. The phagocytic activity of the high-molecular-weight fraction LBP1 (1.21 × 10^6^ Da) in Lycium barbarum polysaccharides (LBPs) was also significantly higher than that of the low-molecular-weight fraction LBP2 (1.25 × 10^5^ Da) [47]. In this study, the molecular weight of polysaccharides extracted by the hot water method was 812.53 kDa, which was 8.13 times that of polysaccharides extracted by the DES method. This may be an important reason for its stronger ability to induce the phagocytic activity of macrophages.

NO is an intracellular signal transduction molecule released during the immune response of macrophages, which exerts toxic effects on invading pathogenic microorganisms and tumor cells [48]. Compared with the control group (Figure 6C), both the LPS group and the polysaccharide groups significantly stimulated NO release in RAW264.7 cells (*p* < 0.05). At a concentration of 0.25 mg/mL, the NO secretion of the hot water extraction group reached a maximum of 34.14 μmol/L, which was 94.98% higher than that of the DES extraction group. This suggests that polysaccharides extracted by the hot water method have a stronger potential to stimulate NO release.

Furthermore, macrophages can indirectly resist foreign pathogens and stimulants by secreting cytokines [49]. As shown in Figure 6D–E, compared with the blank control group, both polysaccharides significantly stimulated the secretion of cytokines including TNF-α and IL-6 (*p* < 0.05). Interestingly, when the treatment concentration of hot water-extracted polysaccharide reached 0.25 mg/mL, the secretion levels of TNF-α and IL-6 were 1061.15 pg/mL and 1190.13 pg/mL, respectively, which were 1.23-fold and 1.21-fold higher than those of the DES-extracted polysaccharide. It can be concluded that ATSP could induce macrophages to secrete cytokines such as TNF-α and IL-6, indicating that ATSP has the potential to serve as a promising immunostimulant. In conclusion, high extraction efficiency does not equate to high product activity. Although the DES method significantly improved the polysaccharide yield, the biological activity of the DES-extracted polysaccharide—such as promoting the phagocytic capacity of RAW264.7 cells, stimulating NO secretion, and inducing the production of TNF-α and IL-6—was lower than that of the hot water-extracted polysaccharide under the tested in vitro conditions (Figure 6). It should be emphasized that the above conclusions are derived solely from experiments using RAW 264.7 macrophage cell lines, and the relevance of these in vitro findings to in vivo immunomodulatory effects remains to be validated.

It is well known that the immunological activity of polysaccharides is closely associated with their structural characteristics, including molecular weight, monosaccharide composition, and sulfate content [50]. In this study, the polysaccharides obtained by the two extraction methods exhibit multi-dimensional structural differences, specifically manifested by significant variations in molecular weight, monosaccharide composition, sulfate content, and protein content. These structural differences collectively contribute to the divergence in their immunological activity, among which the reduction in molecular weight is a key influencing factor. These differences can be attributed to the intermolecular interactions and ionic behavior inherent in DES, which can decompose long-chain carbohydrate complexes, thereby achieving a higher degree of degradation. For instance, ChCl-LA is an acidic DES that may cause the breakage of polysaccharide chains, resulting in the loss of the high-molecular-weight advantage of hot water-extracted polysaccharides (812.53 kDa, 8.13 times that of DES-extracted ones). This proposed mechanistic explanation—that acidic DES breaks glycosidic bonds and destroys conformational features essential for receptor activation—is a hypothesis inferred from the observed Mw reduction and relevant literature reports, as the present study did not directly measure the higher-order conformations of polysaccharides or their binding affinity with immune receptors. It is worth noting that the relationship between polysaccharide molecular weight and immunological activity is highly structure-dependent: while lower Mw fractions enhance the immunostimulatory activity of certain polysaccharides (e.g., via improved bioavailability and cellular absorption), the opposite trend was observed in the current *A. taxiformis* polysaccharide system, with the hot water-extracted polysaccharides of high molecular weight showing stronger activity. This result further corroborates that the reduction in molecular weight induced by DES extraction may decrease the number of binding sites for immune receptors (such as TLR4 and Dectin-1) on the surface of RAW264.7 macrophages, thereby impairing immune activation efficiency. Simultaneously, differences in monosaccharide composition and sulfate content may also synergistically regulate this process. This receptor-mediated regulatory pathway is a plausible mechanism, but remains speculative; confirmation would require further experiments such as receptor-specific blocking assays or gene silencing techniques targeting TLR4 and Dectin-1.

This is supported by Guo et al. (2021), who reported that this phenomenon might occur because the intermolecular and intramolecular interactions of DES can disrupt the complex structure of polysaccharides, leading to their depolymerization into smaller molecular units [51]. Additionally, Gu et al. (2023) pointed out that due to the combined effects of intermolecular hydrogen bonds, van der Waals forces, and electrostatic interactions, DES exhibits a stronger binding capacity for polysaccharide structures, which significantly improves dissolution efficiency [52]. However, it is noteworthy that the strong interaction effect in DES may not only disrupt the hydrogen bond networks between polysaccharides and cell walls, but also irreversibly break their glycosidic bond linkages, resulting in damage to the conformational structures or functional domains required for immune activation. Taken together, ATSP can exert immunomodulatory effects by enhancing the phagocytic function, stimulating NO secretion, and inducing the secretion of TNF-α and IL-6 in RAW264.7 cells. Moreover, hot water-extracted polysaccharides exhibit more significant activity due to the retention of a more intact high-molecular-weight structure and stable chemical composition (including monosaccharides and sulfate groups). This suggests that the extraction method for polysaccharides needs to balance yield and structural integrity, providing a theoretical reference for the efficient utilization of marine polysaccharides.

## 4. Conclusions

This study outlines an efficient and green extraction technology for polysaccharides from *A. taxiformis*: among 12 DESs, ChCl-LA was screened as the optimal solvent. After optimization via RSM, the yield of crude polysaccharides reached 11.28% ± 0.50%, which was 1.5 times higher than that of the traditional hot water extraction method. The obtained polysaccharide was acidic, mainly composed of galactose (89%), with a weight-average molecular weight (Mw) of 99.88 kDa (notably lower than the 812.53 kDa of hot water-extracted polysaccharides). DFT and MD simulation analyses indicated that ChCl-LA easily forms more hydrogen bonds with galactose, significantly improving its solubility. In vitro cell experiments suggested that both polysaccharides exert immunomodulatory effects by enhancing RAW264.7 cell phagocytosis, stimulating the secretion of NO and cytokines including TNF-α and IL-6; however, the immunostimulatory activity (e.g., macrophage activation) of DES-extracted polysaccharides was significantly diminished. This study reveals a trade-off between extraction efficiency and immunological activity of *A. taxiformis* polysaccharides: ChCl-LA improves yield, but reduces Mw and impairs macrophage activation. Future work should focus on optimizing DES compositions and extraction conditions (e.g., milder acidity, shorter exposure time) to preserve polysaccharide bioactivity while maintaining DES’s green advantages. Overall, optimized ChCl-LA (for bioactivity retention) is a promising alternative to improve the efficiency of polysaccharide extraction. This study provides green technical support for the high-value utilization of red alga *A. taxiformis* and lays a foundation for polysaccharide’s application in immune-related diseases.

## Figures and Tables

**Table 1:** Analysis of variance for the significance of regression model coefficients.

Source of Variation	Sum of Squares	Degrees of Freedom	Mean Square	F Value	*p* Value	Significance
Model	17.67	9	1.96	58.20	<0.0001	**
A—Solid-to-liquid Ratio	1.17	1	1.17	34.69	0.0006	**
B—Extraction Temperature	0.3872	1	0.3872	11.48	0.0116	*
C—Ultrasonic Power	1.07	1	1.07	31.59	0.0008	**
AB	0.0441	1	0.0441	1.31	0.2905	
AC	0.0729	1	0.0729	2.16	0.1850	
BC	2.37	1	2.37	70.29	<0.0001	**
A^2^	3.18	1	3.18	94.35	<0.0001	**
B^2^	0.0879	1	0.0879	2.61	0.1505	
C^2^	8.48	1	8.48	251.47	<0.0001	**
Residual	0.2362	7	0.0337			
Lack of Fit	0.1635	3	0.0545	3.00	0.1583	
Pure Error	0.0727	4	0.0182			
Total	17.19	16				

Notes: * indicates a significant difference (*p* < 0.05), and ** indicates a highly significant difference (*p* < 0.01).

## Data Availability

The original contributions presented in this study are included in the article/Supplementary Materials. Further inquiries can be directed to the corresponding author.

## Supplementary Materials

The following supporting information can be downloaded at: https://www.mdpi.com/article/10.3390/foods15030438/s1, Table S1: 12 types of deep eutectic solvents (DESs) used in this experiment. Table S2: Analysis of factors and levels in response surface experiment. Table S3: Experimental design and results of RSM for A. taxiformis sulfated polysaccharide yield.
