# Supplementary material for: Deep Eutectic Solvent Ultrasonic-Assisted Extraction of Polysaccharides from Red Alga Asparagopsis taxiformis: Optimization, Characterization, Mechanism, and Immunological Activity in RAW264.7 Cells
Source: Foods. 2026 Jan 25;15(3):438. doi: 10.3390/foods15030438 (PMC12896575; doi:10.3390/foods15030438)
Supplement: Supplementary file 1 [file foods-15-00438-s001.zip › foods-4062910-supplementary.pdf]

supplementary material

Table S1 12 types of deep eutectic solvents (DESs) used in this experiment

DESs-number	HBA	HBD	Molar ratio (Mol/mol)
DES-1	Choline Chloride	Glucose	1:1
DES-2	Choline Chloride	Urea	1:2
DES-3	Choline Chloride	Citric Acid	1:1
DES-4	Choline Chloride	Lactic Acid	1:2
DES-5	Choline Chloride	Glycerol	1:2
DES-6	Betaine	Glycerol	1:2
DES-7	Choline Chloride	Ethylene Glycol	1:2
DES-8	Choline Chloride	Absolute Ethanol	1:1
DES-9	Betaine	Urea	1:2
DES-10	Betaine	Ethylene Glycol	1:2
DES-11	Betaine	Propylene Glycol	1:3
DES-12	Choline Chloride	Lactic Acid +Ethylene Glycol	1:2:2

Table S2 Analysis of factors and levels in response surface experiment

Level	Factor A: Solid-liquid ratio (g/mL)	Factor B: Extraction temperature (℃)	Factor C: Ultrasonic power (W)
-1	1:25	75	300
0	1:30	85	350
1	1:35	95	400

Table S3 Experimental design and results of RSM for *A. taxiformis* sulfated polysaccharide yield

Run Number	Solid-to-liquid Ratio (g/mL)	Extraction Temperature (°C)	Ultrasonic Power (W)	Polysaccharide Yield (%)
1	30	75	300	9.34
2	35	85	300	8.83
3	35	85	400	8.11
4	30	85	350	11.23
5	30	75	400	10.41
6	25	75	350	10.78
7	25	95	350	10.24
8	25	85	300	9.91
9	35	75	350	9.85
10	30	85	350	11.35
11	25	85	400	8.65
12	30	95	300	10.33
13	30	85	350	11.14
14	30	95	400	8.32
15	30	85	350	10.99
16	35	95	350	9.73
17	30	85	350	11.11

Correlations between physicochemical properties of different DESs (n=12) and polysaccharide yield as well as total sugar content. (The values presented below are the average of three replicate experiments)

	PH	Viscosity	polarity	yield	Total sugar cont
DES-1	4. 81	20	99. 96	0. 0362	0. 6377
DES-2	8. 35	5. 67	103. 59	0. 036	0. 4432
DES-3	0. 62	15. 33	102. 11	0. 0322	0. 6519
DES-4	1. 02	12. 33	102. 11	0. 1188	0. 6283
DES-5	5. 27	15. 67	103. 97	0. 0313	0. 5741
DES-6	6. 36	20. 67	102. 48	0. 0838	0. 4818
DES-7	5. 22	10	101. 38	0. 0518	0. 5512
DES-8	5. 28	4. 67	101. 38	0. 0585	0. 4886
DES-9	8. 55	7	101. 75	0. 0978	0. 4609
DES-10	7. 44	13	100. 32	0. 098	0. 409
DES-11	7. 21	19. 67	102. 48	0. 0942	0. 5717
DES-12	1. 53	12. 33	101. 39	0. 0747	0. 47
